# High-dimensional immune profiling by mass cytometry revealed immunosuppression and dysfunction of immunity in COVID-19 patients

**DOI:** 10.1038/s41423-020-0447-2

**Published:** 2020-04-28

**Authors:** Wenjing Wang, Bin Su, Lijun Pang, Luxin Qiao, Yingmei Feng, Yabo Ouyang, Xianghua Guo, Hongbo Shi, Feili Wei, Xiaogang Su, Jiming Yin, Ronghua Jin, Dexi Chen

**Affiliations:** 1 Beijing Institute of Hepatology, Beijing Youan Hospital, Capital Medical University, 100069 Beijing, China; 2 Beijing Precision Medicine and Transformation Engineering Technology Research Center of Hepatitis and Liver Cancer, 100069 Beijing, China; 3 Center for Infectious Diseases, Beijing Youan Hospital, Capital Medical University, 100069 Beijing, China

**Keywords:** Infection, Cell death and immune response

The outbreak of coronavirus disease 2019 (COVID-19) caused by the new virus SARS-CoV-2 has been announced as a public health emergency of international concern.^1–3^ The clinical features of patients with COVID-19 range from common fever and cough to other rare symptoms, such as diarrhea and nausea. This disease can progress quickly, and 2–3% of patients die within a short time, which is generally due to multiple organ failure.^4–7^ Clinically, COVID-19 patients are classified into mild, moderate, severe, and critical cases.^5–7^ The immune response against SARS-CoV-2 is probably linked to the severity of disease. Recently, Zheng et al.^8^ showed that elevated levels of T-cell exhaustion and reduced functional diversity of T cells in peripheral blood may predict severe progression in COVID-19 patients; however, a more comprehensive understanding of the pathology of SARS-CoV-2 infection remains to be delineated. Here, we profiled immune cellular components using mass cytometry (CyTOF) to analyze the peripheral blood mononuclear cells (PBMCs) from patients with differences in disease progression by comparing with the PBMCs from healthy donors (HDs).

To find evidence of alterations in leukocyte homeostasis, we collected CD45^+^ PBMCs from 12 HDs (*n* = 12) and a varying number of COVID-19 patients with different clinical conditions (mild, *n* = 4; severe, *n* = 5; and critical, *n* = 3; Supplementary Fig. 1a). Among the patients, the average age was 58 years old; 42% were men, and 57.1% were women. A total of 42.9% had chronic diseases, such as hypertension, diabetes, and cardiovascular diseases (Supplementary Table 1). By virtue of CyTOF, we differentiated CD45^+^ PBMCs into 31 clusters using metal-labeled antibodies (Supplementary Table 2) against 18 immune cell surface markers and observed obvious differences in the composition of CD45^+^ PBMC populations in HDs and COVID-19 patients under different clinical conditions (Fig. 1a–c and Supplementary Fig. 1b). Within all of the clusters, we found that CD4, CD8, CD45RA, CD45RO, CD5, CXCR3, and especially CD11b displayed relatively more dynamic expression (Fig. 1b and Supplementary Fig. 1d). Furthermore, we analyzed the percentages of subsets of immune cells represented by each cluster and found the major differences between HDs and patients; while there were no significant differences among the patients of the mild, severe, and critical cases (Fig. 1c and Supplementary Fig. 1c).

**Fig. 1:**
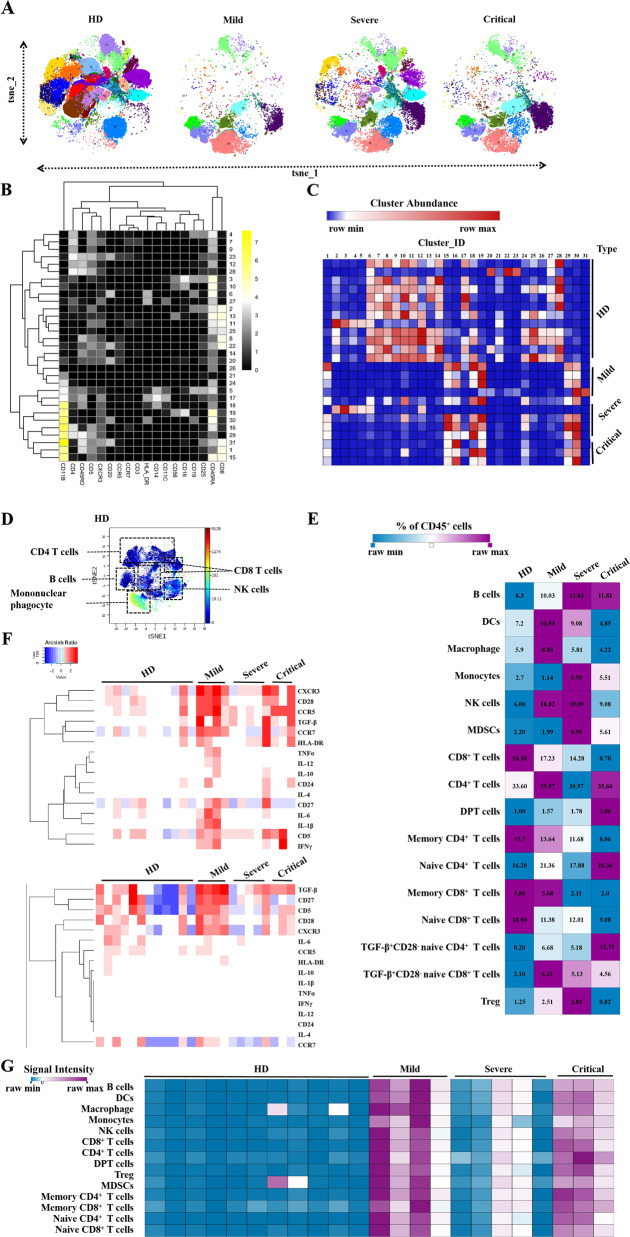
CyTOF-based analysis identified immune cell signatures in peripheral blood of COVID-19 patients. **a** Representative viSNE plot of immune cell populations from HDs and COVID-19 patients. **b** Heatmap of the expression pattern of indicated sorting markers by 31 clusters. **c** Heatmap of the frequency of displayed clusters among HDs and patients. Compared to HDs, clusters 6–14, 17, and 24–28 declined but clusters 15–16, 18–19, and 29–30 increased among patients with all clinical cases. **d** Representative image of immune cell populations identified by CyTOF. **e** The percentage of B cells, DCs, macrophage, monocytes, NK cells, MDSCs, CD8^+^ T cells, CD4^+^ T cells, DPT cells, Tregs, and different CD4^+^/CD8^+^ T-cell subsets are shown in the heatmap. **f** Heatmap of indicated cytokine expression in CD4^+^CD25^+^CD127^-^ Tregs (upper panel), and CD4^+^CD8^+^CD25^−^ DPT cells (lower panel). **g** Heatmap of TGF-β expression in the indicated immune cells in peripheral blood from HDs and patients under mild, severe, and critical conditions. The color key in the heatmap represents the magnitude of expression (**b**), cluster abundance (**c**), signal intensity (**d**, **g**), percentage of CD45^+^ cells(**e**), and arcsinh ratio by normalizing to the control (**f**)

To characterize the heterogeneity of inflammatory immune cells in response to novel coronavirus infection, CD4^+^ and CD8^+^ T cells, B cells, NK cells, and mononuclear phagocytes were further profiled (Fig. 1d). When compared to those in HDs, we found that the proportions of B cells, CD4^+^CD8^+^ double-positive T cells (DPTs), naïve CD4^+^ T cells, and TGF-β^+^CD28^-^ naïve CD4^+^ T cells in infected patients were generally increased, whereas CD8^+^ T cells, regardless of whether they belonged to the effector, naïve, or memory subsets, declined constantly during the progression of infection. Additionally, NK cells, monocytes, myeloid-derived suppressor cells (MDSCs), and regulatory T cells (Tregs) appeared to show the same pattern: they increased during the progression from mild to severe condition but then declined during the progression to critical condition. Moreover, we observed that the proportions of dendritic cells (DCs), macrophages, CD4^+^ T cells, and TGF-β^+^CD28^-^ naïve CD8^+^ T cells were higher in the mild group than in the severe group (Fig. 1e). These data indicated the disturbed homeostasis of immune system in the patients with the dysfunctions of CD8^+^ T cells, DCs, and macrophages that were excessively activated at first and became exhausted thereafter.

It is confirmed that Tregs are crucial to maintain immune cell homeostasis in numerous diseases.^9^ CD4^+^CD25^+^CD127^−^ Tregs (Fig. 1f, upper panel) and CD4^+^CD8^+^CD25^-^ DPT cells (Fig. 1f, lower panel) both have a high capacity to produce cytokines that act as immunosuppressive regulators of leukocyte trafficking.^10,11^ We therefore profiled their expression and found that the levels of functional molecules such as CXCR3, CD28, and TGF-β were obviously higher in the patient groups than in the HD group (Fig. 1f). In particular, increased expression of TGF-β among all the patient groups occurred in multiple types of immune cells (Fig. 1g). Additionally, MDSCs can secrete immunosuppressive cytokines, such as TGF-β and IL-10, to induce regulatory T-cell development, which has been implicated in the regulation of various immune processes in diseases, including infection.^12^ The numbers of T cells and NK cells were reduced in SARS-CoV-2-infected patients, which was probably due to the combinational interactions of these cells. Remarkably, increased infiltration of MDSCs was associated with poor prognosis and resistance to therapies among cancer patients.^13,14^ To investigate the circulating MDSCs may have diagnostic value in the prognosis of COVID-19 patients.

To strengthen the above findings, further experiments might be helpful by increasing the enrolled patients and expanding the used antibodies besides ones included in CyTOF. SARS-CoV-2 has displayed its unique properties during the epidemic although it shares some similarities with SARS-CoV and MERS-CoV. Further revealing the host immune responses against SARS-CoV-2 is one of the crucial steps to find efficient drugs, develop preventive vaccines and finally overcome the worldwide pandemic in the near future.

## Supplementary information

Supplementary material_Revised
