# Supplementary material for: High-dimensional immune profiling by mass cytometry revealed immunosuppression and dysfunction of immunity in COVID-19 patients
Source: Cell Mol Immunol. 2020 Apr 28;17(6):650–2. doi: 10.1038/s41423-020-0447-2 (PMC7186533; doi:10.1038/s41423-020-0447-2)

**Table 1. Clinical characteristics of patients diagnosed with SARS-CoV-2.**

| Baseline variables | All patients | Mild | Severe | Critical |
| --- | --- | --- | --- | --- |
|  | n=12 | n=4 | n=5 | n=3 |
| Characteristics |  |  |  |  |
| Age (year) | 58±15.4 | 45±12.5 | 65±10.0 | 71±4.0 |
| Gender (%) |  |  |  |  |
| Men | 6 (42.9) | 2 (33.3) | 3 (60.0) | 1 (33.3) |
| Women | 8 (57.1) | 4 (66.7) | 2 (40.0) | 2 (66.7) |
| Underlying diseases (%) | 6 (42.9) | 1 (16.7) | 2 (40.0) | 3 (100.0) |
| hypertension | 5 (35.7) | 1 (16.7) | 1 (20.0) | 3 (100.0) |
| Coronary heart diseases | 3 (21.4) | 1 (16.7) | 1 (20.0) | 1 (33.3) |
| diabetes | 2 (14.3) | 1 (16.7) | 0 | 1 (33.3) |
| Other complications (%) | 3 (21.4) | 1 (16.7) | 1 (20.0) | 1 (33.3) |
| Gallstone | 1 (7.1) | 1 (16.7) | 0 | 0 |
| Reflux Esophagitis | 1 (7.1) | 0 | 1 (20.0) | 0 |
| Chronic Kidney Disease | 1 (7.1) | 0 | 0 | 1 (33.3) |
| WBC | 4.81±1.96 | 4.51±1.24 | 4.94±2.47 | 5.20±3.21 |
| N | 3.34±2.09 | 2.56±0.94 | 3.72±2.80 | 4.29±3.01 |
| L | 1.03±0.57 | 1.42±0.53 | 0.85±0.51 | 0.56±0.37 |
| PLT | 234.93±100.1 | 258.70±131.6 | 194.8±89.69 | 254.3±66.88 |
| ALT | 32.50±26.09 | 35.50±39.22 | 33.20±14.74 | 25.33±19.50 |
| AST | 36.00±18.66 | 35.00±20.71 | 35.20±10.92 | 39.33±33.26 |
| TBIL | 11.93±5.43 | 11.00±4.54 | 11.02±3.70 | 15.30±10.28 |
| CK | 127.29±102.65 | 115.20±125.90 | 103.00±36.66 | 192.00±152.4 |
| CREA | 70.00±20.04 | 64.67±14.95 | 64.00±17.85 | 90.67±28.29 |
| CKMB | 1.06±0.83 | 1.09±1.05 | 0.88±0.89 | 1.28±0.52 |
| SpO2 | 96.69±3.04 | 98.67±1.15 | 96.24±2.10 | 93.50±5.00 |

**Table 2. Mass cytometry antibodies panel design.**

| Antigen | Symbol and Mass | Antibody clone | Source |
| --- | --- | --- | --- |
| CD45 | 89Y | HI30 | Fluidigm |
| CD45 | 141Pr | HI30 | Fluidigm |
| CD19 | 142Nd | HIB19 | Fluidigm |
| CD5 | 143Nd | UCHT2 | Fluidigm |
| CCR5 | 144Nd | NP-6G4 | Fluidigm |
| CD4 | 145Nd | RPA-T4 | Fluidigm |
| CD45RA | 146N | HI100 | biolegend |
| CD20 | 147Sm | H1 | Fluidigm |
| CD14 | 148Nd | 134620 | R&D |
| CD56 | 149Sm | NCAM16.2 | Fluidigm |
| CD11c | 150Nd | Bu15 | biolegend |
| CD16 | 151Eu | 3G8 | biolegend |
| TNFα | 152Sm | MAb11 | Fluidigm |
| CD62L | 153Eu | DREG-56 | Fluidigm |
| IL-1β | 154Sm |  | Abcam |
| CD27 | 155Gd | L128 | Fluidigm |
| CXCR3 | 156Gd | G025H7 | Fluidigm |
| IFN-γ | 158Gd | B27 | Fluidigm |
| CCR7 | 159Tb | G043H7 | Fluidigm |
| CD28 | 160Gd | CD28.2 | Fluidigm |
| CD25 | 161Dy | BC96 | biolegend |
| CD8 | 162Dy | RPA-T8 | Fluidigm |
| TGF-β | 16Dy | TW46H10 | Fluidigm |
| CD45RO | 164Dy | UCHL1 | Fluidigm |
| IL-12 | 165Ho |  | Abcam |
| IL-10 | 166Er | JES3-9D7 | Fluidigm |
| IL-6 | 167Er | MQ2-13A5 | Biolegend |
| CD206 | 168Er | 15-2 | Fluidigm |
| CD24 | 169Tm | ML5 | Fluidigm |
| CD3 | 170Er | UCHT1 | Fluidigm |
| CD68 | 171Yb | Y1/82A | Fluidigm |
| CD45 | 172Yb | HI30 | Biolegend |
| HLA-DR | 173Yb | L243 | Fluidigm |
| IL-4 | 174Yb | MP4-25D2 | biolegend |
| CD127 | 176Yb | 40131 | R&D |
| CD11b | 209Bi | ICRF44 | Fluidigm |

**Supplementary Figure 1.** CyTOF-based analysis identified immune cell signatures in peripheral blood of COVID-19 patients. A) Gates in each plot show the events of the indicated stained protein by CyTOF and CD45 debarcod. B) viSNE plots show the compartment of 2.4 x 10^5^ immune cells from peripheral blood, which were colored according to either type (left) or cluster (right). C) Boxplots display the frequency of displayed clusters across indicated conditions. D) Expression patterns of immune cell subsets in PhenoGraph analysis.


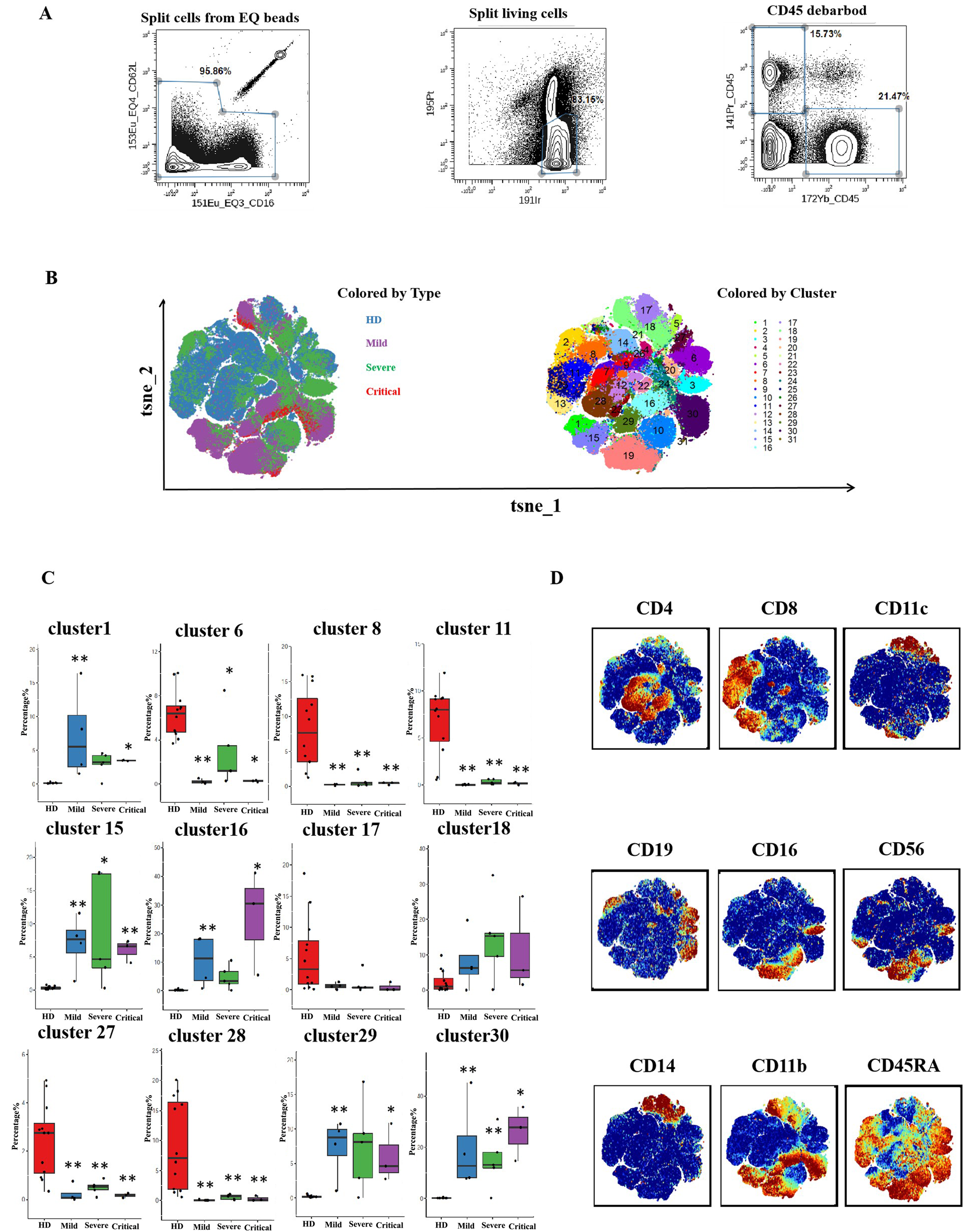

Supplement: Supplementary file 1 — Supplementary material_Revised [file 41423_2020_447_MOESM1_ESM.docx]
